# Advances in Amylases—What’s Going on?

**DOI:** 10.3390/molecules28217268

**Published:** 2023-10-25

**Authors:** Štefan Janeček

**Affiliations:** 1 Laboratory of Protein Evolution, Institute of Molecular Biology, Slovak Academy of Sciences, SK-84551 Bratislava, Slovakia; stefan.janecek@savba.sk; 2 Institute of Biology and Biotechnology, Faculty of Natural Sciences, University of SS. Cyril and Methodius, SK-91701 Trnava, Slovakia

## 1. Introduction

With regard to the CAZy database ([1]; http://www.cazy.org/ accessed on 14 October 2023) classifying carbohydrate-active enzymes into their sequence-based families, and based on the obtained knowledge, it is reasonable to consider that α-amylase enzyme specificity is present in four CAZy glycoside hydrolase (GH) families: GH13, GH57, GH119, and GH126 [2]. In other words, currently, of the 186 GH families in total [1], 4 may be considered α-amylase families [2]: (i) GH13—the main and the largest α-amylase family; (ii) GH57—the second and smaller α-amylase family; (iii) GH119—a very small family related to GH57; and (iv) GH126, in which the presence of pure α-amylase specificity has still not been proven definitively. Particularly, the two former α-amylase families, GH13 and GH57, cover, in addition to α-amylase, most of the numerous amylolytic enzymes, such as α-glucosidase, pullulanase, amylopullulanase, isoamylase, cyclodextrin glucanotransferase, 4-α-glucanotransferase, α-glucan branching enzyme, α-glucan debranching enzyme, trehalose synthase, sucrose isomerase, etc. [3], while two other “amylases”, β-amylase and glucoamylase, constitute their own families, GH14 and GH15, respectively [1,4]. Since they both employ the inverting reaction mechanism, which is the opposite of the retaining mechanism of α-amylase, they have fallen somewhat outside mainstream research focused on starch hydrolases and related amylolytic enzymes that share their sequence-structural aspects with α-amylase [2,3,5,6,7,8,9,10]. It is important to point out, however, that the GH13 family forms a CAZy clan, GH-H, with families GH70 and GH77, which mainly comprise glucansucrases and amylomaltases, respectively [3,11,12]. With respect to the existence of clans of GH families that represent a higher level of hierarchy connecting the originally independent families [13], toward the lower level of hierarchy, a division into subfamilies inside a GH family has also been established [14]. As yet, of the four α-amylase families, only family GH13 has been officially divided into subfamilies. This was first carried out in 2006 via the creation of 35 GH13 subfamilies [15], the one most recently defined being subfamily GH13_47 [16]. Finally, in any efforts devoted to understanding amylolytic enzymes, their non-catalytic starch-binding domains (SBDs) should not be forgotten [17]. These SBDs have also been classified in the CAZy database as the so-called carbohydrate-binding module (CBM) families [1,14]. Thus, of the 98 CBM families created so far, 16 may be considered to represent SBDs: 20, 21, 25, 26, 34, 41, 45, 48, 53, 58, 68, 69, 74, 82, 83, and 98 [16,17].

The scope of amylolytic enzymes, grouped especially in the main α-amylase clan, GH-H, but also in the additional α-amylase families GH57, GH119, and GH126 [2], has been exhibited as enormous during recent decades. This is even reflected in the establishment of a series of international symposia “*on the α-amylase family*” in 2001 called ALAMYs (http://imb.savba.sk/~janecek/Alamys/ accessed on 14 October 2023), which are dedicated, in fact, to all aspects of these enzymes. The ALAMY symposia are organized once every three years, traditionally at the Smolenice Castle near Bratislava in Slovakia. Until now, the last meeting of this series—The Eighth Symposium on the Alpha-Amylase Family (ALAMY_8) grouping together “*amylase-positive researchers*”—was held on 9–13 October 2022. In addition to basic information about ALAMY_8′s sessions, topics and main speakers, those interested in reading some news posted during the symposium can visit the social network X (https://twitter.com/8_alamy accessed on 14 October 2023). I, as the main organizer of ALAMYs symposia, have been extremely pleased about the opportunity to arrange—based on ALAMY_8—a Special Issue of the journal *Molecules* (MDPI) entitled “*Advances in Amylases*”. It was decided that the Special Issue would be open not only to attendees of ALAMY_8, but also to all those who might have a relevant contribution of broader importance to this particular research field. This Special Issue, “*Advances in Amylases*”, comprising thirteen papers—1 review and 12 original articles—has thus been arranged in an effort to present a state-of-the-art collection of highly specific stories focused on amylolytic enzymes classified in the α-amylase GH families to the greatest extent possible.

## 2. The Special Issue “*Advances in Amylases*”

The first part, consisting of four papers, covers amylolytic enzymes in general. Beginning with a *Review* by Blennow, Svensson, and co-workers (contribution 1), various aspects of the interfacial enzyme hydrolysis of native starch granules are discussed emphasizing kinetic approaches in terms of the current understanding of these processes. Then, three original articles follow. Firstly, Zinck et al. (contribution 2) demonstrate the use of sodium hypochlorite to achieve efficient irreversible α-amylase inactivation in the enzymatic processing of raw potato starch. Next, Bai and co-workers (contribution 3) describe the preparation of rice flour with a higher protein content using a granular starch hydrolysing enzyme. Finally, Gänzle and co-workers (contribution 4) deliver results obtained via the cloning of the α-glucan branching enzyme from a swine intestinal metagenome and elucidate its potential role in the formation of branched α-glucans from starch.

The next part of the Special Issue comprises six original articles that are all aimed at individual GH13 subfamilies. In the first one, focused on GH13_9, Geiger and co-workers (contribution 5) present a new three-dimensional structure of the *Escherichia coli* α-glucan branching enzyme complexed with maltooctaose, suggesting a possible mechanism for transfer chain specificity also involving surface binding sites. Next, Møller and co-workers (contribution 6) explore the transglycosylation activity of GH13_13 limit dextrinase from barley using two approaches with different combination of donors and acceptors. Then, a long-awaited tertiary structure of the α-amylase from *Drosophila melanogaster* from subfamily GH13_15 is described by Aghajari, Da Lage and co-workers (contribution 7) together with its biochemical characterization and a discussion of its evolutionary implications. The next study by Woo, Park, and co-workers (contribution 8) is of extremely significant interest since it reveals a circular permutation in the domain C—with a C–A–B–A–C domain order—in the structure of the periplasmic α-amylase MalS from *E. coli* of subfamily GH13_19. The subsequent protein design process described by Saburi, Mori, and co-workers (contribution 9) demonstrates the role of Asn258 in the substrate specificity and transglucosylation activity of *Bacillus* sp. AHU2216 α-glucosidase from subfamily GH13_31. The papers focused on GH13 subfamilies are followed by a detailed in silico analysis of a group of amylolytic enzymes represented by cyclomaltodextrinase from *Flavobacterium* sp. no. 92; this analysis being conducted by Mareček and Janeček (contribution 10), with the result of creating the novel subfamily GH13_46 for this group of enzymes possessing a CBM-like domain at their N-terminus and the segment MPDLN in their fifth conserved sequence region.

The last part of this Special Issue contains three original articles, two of which concern SBDs from the family CBM20 and the remaining one pertaining to a cyclo/maltodextrin-binding protein. First, Punt, Sidar, and co-workers (contribution 11) describe the GH13 α-amylase from *Aspergillus niger* with CBM20 from GH15 *A. niger* glucoamylase artificially designed at its N-terminus, achieving the improved binding and processing of various starches. Then, Svensson, Møller, and co-workers (contribution 12) demonstrate the impact of the CBM20 SBDs originating from GH77 *Solanum tuberosum* 4-α-glucanotransferase and GH15 *A. niger* glucoamylase fused to the N-terminus of the family GH77 *Thermoproteus uzoniensis* amylomaltase on its activity and starch product structure. In the final paper, structure-function relationships are elucidated by Serrano-Posada, Centeno-Leija, and co-workers (contribution 13) in relation to a novel cyclo/maltodextrin binding protein playing a role in the cyclodextrin ABC importer system in Thermoanaerobacterales.

## 3. Conclusions

In conclusion, it should be said that such a single Special Issue of a journal cannot cover all particular aspects within any research field that would deserve to be covered; this would simply be impossible. Nevertheless, I am confident in the top-quality papers presented here covering various hot topics from the research field of amylolytic enzymes.

Finally, I would like to express my sincere appreciation to MDPI Managers responsible for the journal *Molecules* for their trust in this endeavour and for giving me the privilege to be the Guest Editor of this Special Issue. I would also like to thank both contributing authors and respected reviewers whose joined efforts gave rise to this collection of articles. Last but not least, I would like to mention and thank the generous help and support of my MDPI Editorial Assistant, Mr. Harry Zhong, who took excellent care with technical editorial matters during the preparation of this Special Issue. Now, please enjoy “*Advances in Amylases*”!
